# General practice nurse perceptions of barriers and facilitators to implementation of best-practice dementia care recommendations—a qualitative interview study

**DOI:** 10.1186/s12875-024-02401-9

**Published:** 2024-05-02

**Authors:** Caroline Gibson, Dianne Goeman, Dimity Pond, Mark Yates, Alison Hutchinson

**Affiliations:** 1 University of Newcastle, School of Medicine and Public Health, Callaghan, NSW 2308 Australia; 2 Deakin University, School of Medicine, Geelong, Australia; 3 Deakin University, School of Nursing and Midwifery, Burwood, Australia; 4 Grampians Health, Ballarat, Australia; 5 Faculty of Medicine, Nursing and Health Sciences, The Alfred Centre, Melbourne, Monash University, Clayton, Australia; 6 University of Tasmania, Wicking Dementia and Teaching Centre, Hobart, Australia

**Keywords:** General practice nurses, Dementia, Care planning, Primary care, Best-practice recommendations

## Abstract

**Introduction:**

With an aging population and a growing prevalence of people living with dementia, the demand for best-practice dementia care in general practice increases. There is an opportunity to better utilise the nurse role within the primary care team to meet this increasing demand in the provision of care for people living with dementia. However, general practice nurses have limited knowledge in the provision of best-practice care for people living with dementia and their carer(s). A number of best-practice dementia care recommendations contained in the Australian Clinical Practice Guidelines and Principles of Care for People with Dementia have been identified as highly relevant to the role of the general practice nurse.

**Aims:**

To explore general practice nurses’ perspectives on published best-practice dementia care recommendations relevant to their role and identify barriers and facilitators to their implementation into clinical practice.

**Methods:**

Thirteen Australian general practice nurses took part in this qualitative interview study. The research questions for this study were addressed within a paradigmatic framework of social constructionism. Data were transcribed verbatim and thematically analysed.

**Results:**

There was a high level of agreement between general practice nurses that the recommendations were important, reflected best-practice dementia care and were relevant to their role. However the recommendations were perceived as limited in their usefulness to nurses’ clinical practice due to being too vague and lacking direction. Four main themes were identified describing barriers and facilitators to operationalising best-practice dementia care.: creating a comfortable environment; changing approach to care; optimising the general practice nurse role and working collaboratively. Nine sub-themes were described: physical environment; social environment; complexity of care; care planning for the family; professional role and identity, funding better dementia care, education, networking and resources; different roles, one team; and interagency communication.

**Conclusion:**

This study identified several factors that need addressing to support general practice nurses to integrate best-practice dementia care recommendations into daily clinical practice. The development of interventions needs to include strategies to mitigate potential barriers and enhance facilitators that they perceive impact on their delivery of best-practice care for people living with dementia and their carer(s). The knowledge gained in this study could be used to develop multi-faceted interventions informed by theoretical implementation change models to enable the general practice nurse to operationalise best-practice dementia care recommendations.

## Introduction

Dementia is a complex chronic disease and a leading cause of disability among older people [1]. Worldwide, dementia is expected to affect almost 50 million people, and this number is predicted to double by 2050, placing significant pressure on healthcare systems [2]. To better meet the chronic and cumulative dementia healthcare needs [3], there is a growing global health policy commitment to a more proactive approach to the diagnosis and management of people living with dementia (PLWD) in the primary care setting [1].

Nurses working in general practice, henceforth referred to as general practice nurse (GPN), are significant contributors in the delivery of primary care [4]. It is well established that GPN interventions in the management of chronic disease lead to cost effective positive health outcomes [5]. The proactive, planned and person-centred principles of chronic disease management [6] align with best practice principles of dementia care [7]. This suggests that chronic disease management models of care can provide a framework for the provision of nursing care to PLWD and their carer(s) [8, 9]. However, as GPNs have limited knowledge in the recognition of cognitive impairment and care planning for PLWD [10], practical guidance could assist them to increase their capability to better meet the diverse and complex needs of PLWD and their carer(s) [11]. The most effective models of care are informed by evidence-based clinical practice guidelines customised for the intended end-user [12]. A Delphi study of GPNs’ perceptions of the Australian Clinical Practice Guidelines and Principles of Care for People with Dementia [13] was recently undertaken to identify the recommendations that were of high relevance to their role. [14].

While establishing the most relevant guidelines to the GPN role is important, more than guidelines are needed to improve GPNs’ provision of dementia care [15, 16]. For GPNs to successfully operationalise best-practice dementia care recommendations in their daily practice, effective implementation strategies guided by theoretical frameworks [17], need to first consider potential barriers and facilitators [16]. Whilst barriers to optimal dementia care by the primary care team, with a focus on general practitioners, have been well identified [1, 3, 18–20], few studies have investigated barriers and facilitators specific to the GPN provision of dementia care [11]. To our knowledge this paper is the first to report on a qualitative study that explores the barriers and facilitators perceived by GPNs to impact on their delivery of published best-practice care recommendations for PLWD and their carer(s).

## Methods

### Research aim

In order to better understand the factors influencing GPNs’ implementation of best-practice dementia care recommendations, the aims of the study were to:

1. Gain greater insight into GPNs’ perspectives on the recommendations in relation to their delivery of dementia care.
2. Identify barriers and facilitators to GPNs’ implementation of the recommendations in the General Practice setting.

### Study design

The research questions for this study were addressed within a paradigmatic framework of social constructionism. Social constructionism theory is concerned with the processes by which people describe, explain, or account for the world in which they live [21]. The theory of social constructionism posits that much of what individuals perceive as 'reality' is actually the outcome of a dynamic process of construction influenced by social conventions and structures [22]. This theory offers a broad lens to explore the ‘reality’ of the nurse provision of dementia care by understanding GPN experience and perceptions of their role and perceived barriers and enablers in provision of best practice dementia care.

A qualitative design with semi-structured interviews was used to explore the views of GPNs in relation to the research questions. Semi-structured interviews have the potential to provide a deeper understanding of participant experience than would be obtained from quantitative methods [23]. This approach also allowed for the study participants to raise topics important to them that may not have previously been thought of as pertinent by the research team [24]. The consolidated criteria for reporting qualitative research (COREQ) checklist [25] was used for reporting the research.

### Participants

A purposive sample of Australian GPNs were recruited. The method described by Francis et al. [26] was used to determine an adequate sample size. Initially, 10 interviews were conducted. Sampling ceased when there were three consecutive interviews without additional themes identified. At the point when no new themes or codes were generated, data saturation was deemed to have been reached [27].

### Recruitment

Participants were recruited through promotion of the study on Australian primary care nursing organisations’ websites and social media. These organisations included the Australian Primary Health Care Nurse Association and Practice Nurse networks. Interested GPNs contacted the primary researcher using the email included in the study promotion. The primary researcher forwarded the participant information and consent form (PICF) to each potential participant. On return of the signed consent form an interview time was arranged. As described in the study promotion, each study participant received a $100 gift card in appreciation of their time and contribution to the study.

### Data collection

The interviews were conducted by the primary researcher, a PhD candidate and registered nurse in primary care. The participants were informed the study was part of the primary researchers’ PhD research. No relationship between the interviewer and the participant was established prior to the study commencement. The interviews took place in February and March 2023 using the ZOOM on-line platform [28]. The demographics survey and interview guide were sent to each study participant with the completed demographics survey returned prior to the interview taking place. The interviews ranged between 21 and 61 min, with an average length of 38 min (total minutes = 498 min). The interview discussion was audio-recorded with consent and transcribed verbatim by a professional medical transcription service. The transcripts were not checked by participants as clarification of meaning was sought at the time of discussion.

### Interview guide

**Fig. 1:**
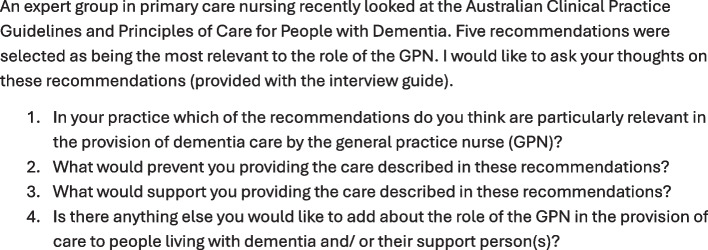
Interview guide

**Fig. 2:**
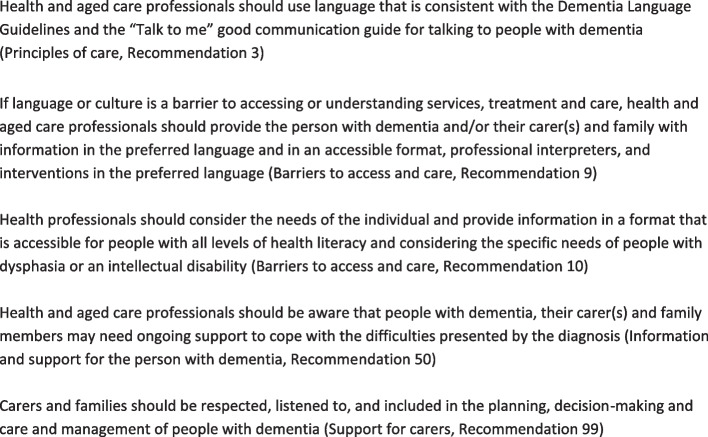
Recommendations highly relevant to the GPN role contained in the Australian Clinical Practice Guidelines and Principles of Care for People with Dementia

The interview guide (Fig. 1) was provided to participants prior to the interview so they had time to consider the five recommendations (Fig. 2) and the guiding questions. The questions were designed by the authors to probe participant perspectives on topics related to the research question.

### Data analysis

The data were coded and analysed using Braun and Clarke’s six steps of thematic analysis; familiarisation, generating codes, constructing themes, revising and defining themes and reporting [27]. All study authors read the transcripts and met to discuss initial impressions of the data in the context of the research aims. Following this, two authors (CG and DG) developed the initial coding framework according to deductive codes identified from study aims and interview guide. CG and DG then independently conducted deductive and inductive coding of three interviews and met again to note any new codes and establish agreement in the final analytic coding framework. The transcribed interviews and codes were entered into the NVivo11 qualitative software [29]. CG and DG then independently coded all data by systematically going through each transcript, highlighting meaningful salient text and attaching an appropriate code from the final analytic coding framework. When new concepts emerged, the coders met to discuss and propose additional inductive codes. Any discrepancies were resolved via consensus The transcripts were reviewed to apply new codes. Each code was noted as either ‘barrier’ or ‘facilitator’, depending on the context in which the code occurred. An example of the coding framework is described in Table 1.

**Table 1:** An example of the coding framework used to arrive at themes

Theme	Sub-theme	Example codes	Example text
Working collaboratively	Different roles, one team	General practice hierarchy (barrier) Nurse lack of autonomy (barrier) Professional respect GP and GPN trust each will do their role (enabler)	*“so I'm very lucky where I am. I've got great GPs and they're very invested in their patient care, and they do take what I say not just, oh, she's just the nurse, and she’s trying to just make me busy or do something, find something that’s not there. They actually respect my position, and my knowledge, and experience and say, oh, okay, if that’s what you think let’s bring him back next week, and let’s do a memory test and let’s take it from there” (GPN 6)*
Optimising the GPN role	Funding better dementia care	General practice is a business (barrier) Funding model does not work for dementia care (barrier) Nurse the best person to provide the care (enabler), but no renumeration attached to nurse care (barrier) Funding nurse-led care (enabler)	*I think the role of the practice nurse could be used a lot better, if there was just better remuneration for it, because obviously you’ve got to bring the money in somehow. (GPN 13)* *“funding is an issue and practice nurses, we don’t get that funding and there’s talk of different codes and things coming in. At moment the doctors or the business is needing us to do so much clinical stuff. If there was more funding so that we could spend that time with people to help set them up. (GPN 7)*
Changing approach to care	Care planning for the family	Funding does not allow including the family/ carer(s) (barrier) Carer support and education(enabler) Value the carer (enabler) Balancing needs (enabler) Recognising challenges for carers(enabler)	*I think that in general practice, we just need to be more supportive of the family members and the cost that it is to try and keep them in the community, and particularly the mental health surrounding it” (GPN 4)* *“Being understanding, I suppose, to the situation. That everything is going to change in the next couple of years for that person and their family and to offer the best support that we can” (GPN 7)* *It's very important that the nurse be respectful or from both the angles… that is important to explain to the support persons why the patient is feeling so – and it's totally normal for someone newly diagnosed or going through a new health need in their life, to go through this. How the support person can connect and to empathise and to slowly take it. It's very important (GPN 2)*

The data analysis phase was designed to maximise trustworthiness and authenticity. Using a systematic approach to develop the coding framework provided rigour with inter-rater reliability testing [30] and an auditable trail of evidence for data transparency enhanced the credibility of findings [31]. The primary researcher (CG) maintained a diary of subjective involvement in the study for reflexive analysis with the aim of reducing researcher bias in the data analysis, acknowledging her own subjectivity as a nurse with GPN experience and the influence that this may have had on the study.

### Patient and public involvement

There was no patient or public involvement in this study. However, consumers were involved in the development of the Australian Clinical Practice Guidelines and Principles of Care for People with Dementia [13] from which the five recommendations discussed by the study participants were drawn.

### Ethics approval

This project was approved by the University of Newcastle’s Human Research Ethics Committee, Approval No. H – 2022 0363.

## Results

### Participants

Thirteen GPNs participated in the study (Table 2). The participants were all female, aged between 30 and 69 years. Ten (77%) participants had 6 years or more experience as a general practice nurse. All participants had experience in chronic disease management. Ten (77%) participants self-reported participating in dementia training, although the type of training was not specified. All Australian states were represented except Tasmania. The two territories were not represented.

**Table 2:** Participant characteristics

	(***N* = 13) n**
**Age (years)**	
30–39	5
40–49	2
50–59	4
60–69	2
**Gender**	
Female	13
**Years of PN experience (years)**	
2–5	2
6–10	7
Over 10 years	4
**Chronic disease management experience**	13
**Attended a dementia training program**	10
**Location of primary place of work as PN**	
Victoria	4
NSW	2
QLD	3
SA	1
WA	3

### GPNs’ perceptions of recommendations overall

Overall, GPNs thought there was a great deal of overlap between the five recommendations and that all were important in delivering care to people living with dementia. Some GPNs chose more than one of the five recommendations as important; some said all were. All the GPNs also described limitations in the usefulness of the recommendations to their clinical practice. For example, they were described as “*long and wordy*" (GPN 1), “*waffly*” (GPN 8), “*fluffy*” (GPN 5) and “*repetitive and overlapping’*”(GPN 10). The GPNs wanted guidelines and recommendations that were easily “a*ccessible* [and] *just make it smart* and *encompass the majority of what we need to be doing”* (GPN 8).

**Fig. 3:**
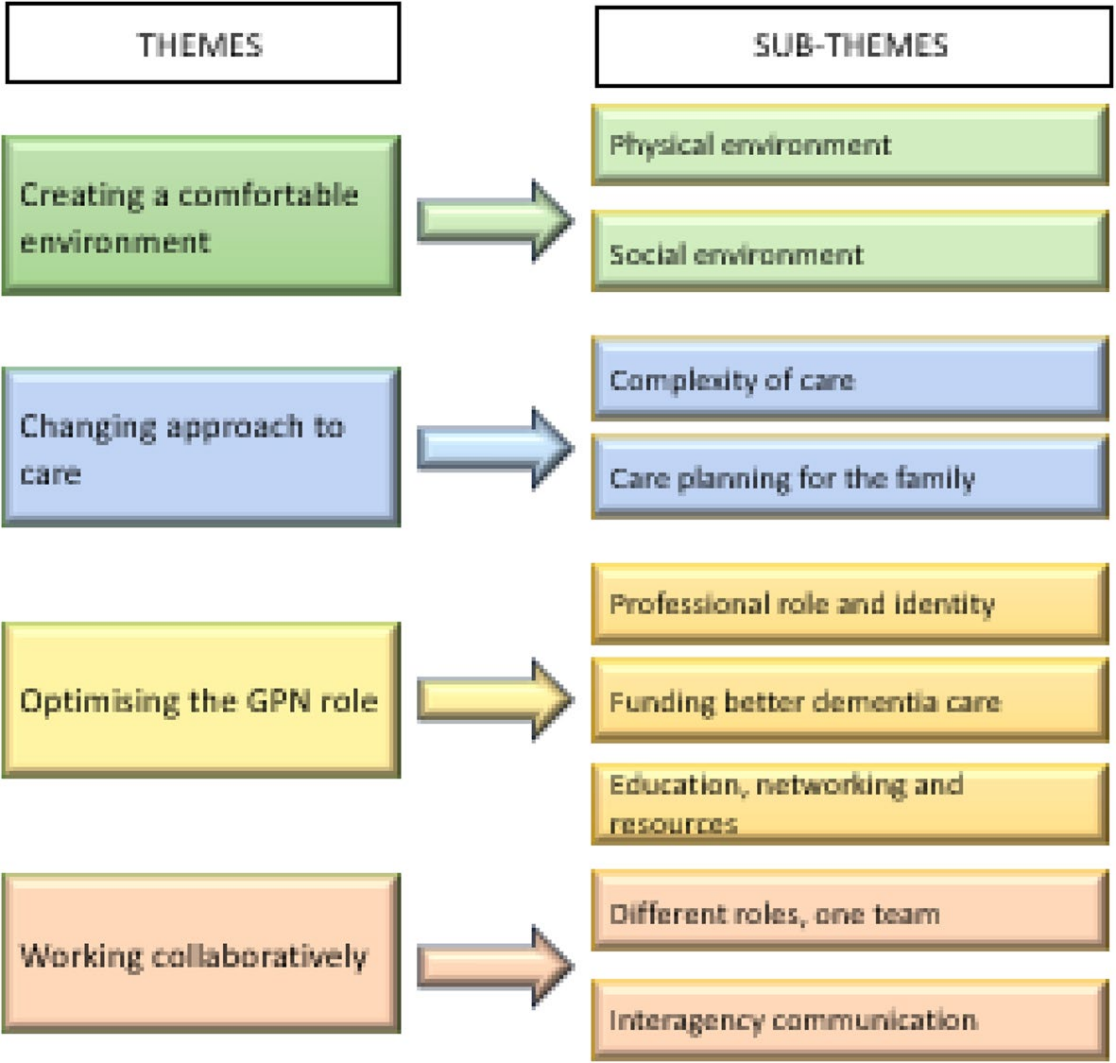
GPN perceptions of the five recommendations—themes and sub-themes

In the interviews, the study participants did not discuss each recommendation separately and specifically. They considered the recommendations in the context of the provision of dementia care overall and the barriers and facilitators often portrayed the same phenomenon from different angles. The four themes and nine sub-themes were identified that described the GPNs perspectives on the factors that impacted on good dementia care provision are presented in Fig. 3.

### Theme Creating a comfortable environment

#### Sub-theme Physical environment

The majority of GPNs highlighted the physical environment of the general practice clinic as a significant barrier to the provision of good dementia care as *“the environment is not set up for people with dementia. It's not a good place to have them in. When we’re even bringing them down into the consulting rooms, even though it's a quiet space, it's very distracting. Even the carers are distracted when they come and see us, so you don't get as much information even out of the carer. I think in primary care, if we could give them more care in the home where they're comfortable and supported, that would be much more ideal than what we're doing at my practice anyway”.* (GPN4).

#### Sub-theme Social environment

In addition to providing a better environment for the provision of care, conducting a home visit was also seen as facilitating better care as it provided contextual understanding of the impact of dementia on the person and carer(s). One nurse described how a home visit provided information that potentially delayed entry into permanent care.

“you can see for yourself about how they're actually managing, and they're happy, because she is at home, … and they’re both managing, and they’ve got services in place … their house is clean, there's no clutter, there's ramps, there's grab rails. …. You can let the GP know. You can stop pushing and pressing for them to go into care because at the moment they're actually managing okay. Why ruin that?” (GPN 13).

The therapeutic nurse-patient relationship was viewed as an important element of creating a supportive environment. The building of rapport and trust enabled the nurse to “*have those difficult conversations that people don’t like to have. But they're important to have, and then if a patient feels that they can open up to you, and trust you, they tell the nurses very different things than they tell the doctors”* (GPN 13)*.* The GPNs felt that a positive nurse-patient relationship also created a safe environment, which could help mitigate the social stigma of dementia and fear and denial of the condition that were described as barriers to the provision of appropriate care. Dementia was described as “*one of those illnesses where it's still hidden. Families don't want to admit that they've got a patient or a relative with dementia. It's hard”* (GPN 8). The GPNs felt that they had the potential to *“make it not a scary subject, because a lot of people link it with dying. It's like blowing the lid off, letting the cat out of the box that it's not a dirty secret, there’s nothing to be ashamed of.”* (GPN 10) It was also felt that it was important to reassure people that the nurse’s role was to provide support to live at home. “*They do have that really preconceived idea, the first thing they say is what—when I say what’s your plan for the next phase of your life? I am not going into a nursing home, that’s the first thing they say to me and I’m going okay, well, that’s not my job to tell you to go to a nursing home. It’s to provide support”* (GPN 9).

### Theme Changing approach to care

#### Sub-theme Complexity of care

All the GPNs described that providing care for PLWD was different to routine care and required a change in approach. However, many of the study participants felt there was a lack of knowledge and skills to provide care to meet the complex needs of PLWD. *“You're just expecting—when you see a name and an age and a medical history on a file, you're expecting a certain flow to go to that consult. When they arrive, it all goes out the window, and then you're floundering wondering how on earth you can help this person in the same way that you would help others. It's really, it's such a difficult presentation to deal with. If you don't have nurses in general practice that understand how to care for someone with dementia, trying to treat them like somebody without it, you're going to miss things. So, I think we do need to alter the way that we care for people with dementia, and I don't think that nurses in general practice are really trained in how to do that”* (GPN 4).

A few GPNs stated that it was essential to consider the impact of dementia when providing chronic disease management for other conditions. *“If you’ve got someone who comes in with diabetes and they have a cognitive impairment, and you wanted to address the diabetes… It changes the way you approach [the care]. The nurses who aren’t so confident don’t want to talk about problems with dementia, but they’re talking about diabetes with someone, and ignore the other parts…you can’t, it’s all rolled into the one patient that you’re dealing with, not the one disease. If they’re doing a care plan and the patient has diabetes and dementia, they need to be on equal status… …so that you don’t miss one and highlight the other one”* (GPN 12).

There was agreement amongst all GPNs that dementia could not be ignored. Early recognition and provision of care was perceived as essential to preventing future problems. *“Prevention is better than cure… prevent the complications of cognitive decline in patients by implementing services and strategies before it gets too bad. When you start to notice that it's mild and patients’ relatives are starting to notice changes too, that is when you intervene, not when it's six months later and they can’t tell you what their name is. Early intervention is key”* (GPN 12).

Identifying health and social care needs and linking people with community support services was perceived as a key element in the provision of dementia care to both the PLWD and their carer(s). “*Being able to navigate where services could be implemented to assist, community-based services, respite, extra nursing support “ (*GPN 13) and *“get those support services in earlier, I say to them, you'll be able to be more independent, more safe, [stay] longer in your house” (*GPN 3). One GPN went so far as to state that *“a good practice or a good practice nurse knows what's available in her community*” (GPN 2). However, all the GPNs acknowledged that it was difficult to know what services were available and this lack of knowledge was a barrier to care. “*If we’ve got the knowledge of how to help and point people in the right direction, it makes such a big difference. But it’s tricky if we’re lacking the knowledge of where to send people to or providing that service*” (GPN 11). GPNs stated that locating services was “*a minefield in itself… each area seems very different about what support is out there”* (GPN 9) and there is a constant need to *“keep up with what services are around your local area, because that's constantly changing”* (GPN 3).

All GPNs talked about the complexity of the health and social care needs associated with dementia as a barrier to care. “*[providing care] covers everything from their ability to drive, their ability to stay at home, their planning for the future, advanced care plans, power of attorney, it’s pretty overwhelming… It can sometimes be a really slow, slow process”* (GPN 3). Taking it slowly, however, was seen as a facilitator as it allowed time to build trust and be respectful of changing needs *“[the GPN] shouldn’t address everything all at once, it should be a step-by-step process, help, when and how they want the help, I can suggest things one year, and the next year I go back, and it's like, have you done anything about that? No, no. It's like, okay. It can sometimes be a really slow, slow process. That's fine, because that's building the trust, it should be a step-by-step process”* (GPN 9).

Continuity of care was perceived as important to regular reviews and providing care to address the changing needs of PLWD. However, the study participants described how a largely part-time and stressed workforce were significant barriers to continuity of care. *“I think the fact that most of us don’t work full-time its difficult following up. Just having different nurses in their care, is often a big barrier. If they’re coming back to see the same nurse, then you can follow-up on their care. Okay, that didn’t work. Let’s try this. What about we put this into place? Let’s tweak that. It’s a huge workload and you’re just trying to get through everyone, get through people, get people sorted. Then if referrals get lost, how do we know? People fall through the cracks”* (GPN 11).

#### Sub-theme Care planning for the family

GPNs described how the provision of care usually focuses on one patient’s needs, the goals they identify and the actions they agree to undertake. However, when providing care for a PLWD it is necessary to involve the carer(s) in care planning and decision-making. One nurse said “*being able to tailor an individualised plan for a family, would be my ideal world situation”* (GPN 6). Part of the care provision for the PLWD was to provide support to the carer to maintain their own health and well-being particularly given the carer role in enabling the PLWD to remain living in the community as their cognitive decline progresses.

“There needs to be a more detailed assessment ongoing evaluation of both them and how their carers are coping, because it gets missed until there’s a big crash and burn, … because if you don’t care for the carer the house is going to fall apart. It will collapse” (GPN 12).

### Theme Optimising the role general practice nurse

#### Sub-theme Professional role and identity

All GPNs in the study perceived the nurse to be the best primary care health professional to provide dementia care. *“We are perfectly suited. We already know [the patient and family], we’ve got a rapport … they appreciate my effort, and they sense the caring. They will come and see me where they may not see your experts, because it's all too hard or intimidating. You do have a knowledge base, everyone is so lost and frightened and scared, just even if you don't do jack except be there and say, oh this must be really unsettling for all of you, or wow, you're doing a fantastic job. You have done something for that person already, or by people carer and the person. The general practice nurse is totally underutilised, they're in the perfect place. They have the knowledge, they have the rapport, they have the skills. You need to use them pretty much”* (GPN 5).

The GPNs felt that part of their value in dementia care was that, unlike the doctor, they could provide more holistic psychosocial care *“with a GP, it's time pressured…that whole psychosocial aspect doesn’t ever get met, or it's pushed to the back and it's like, okay, we’ll deal with that later. That later, never comes. So, I know, as a nurse, I do a lot of that listening to the family members. The doctor is just there to write my scripts and send me for referrals, and fix me, but the nurse will take care of me. So, I think it's the value of having a nurse within a general practice is huge. I would say for any patient that has dementia, is just to have regular reviews with the nurse, we can prevent hospitalisations”* (GPN 13).

The majority of nurses discussed a lack of empowerment as a health professional, which was perceived as demoralising and a barrier to provision of dementia care. *“If it was more nurse led, I believe the nurses would actually be more inclined to follow up and do something on it. But we make these recommendations to the doctor and expect that they follow up* on it. *The nurse does all of this work, makes recommendations to the doctor, and then the doctor goes, okay, well, all right, we’ll order you a blood test, okay, see you next week. Yeah. It's so frustrating. Then the nurses just go, well, what's the point in doing all of this work?”* (GPN 6). One nurse described how being respected professionally facilitated care. “*I'm very lucky where I am. I've got great GPs and they do take what I say not just, oh, she's just the nurse, and she’s trying to just make me busy or do something, find something that’s not there. They actually respect my position, and my knowledge, and experience and say, oh, okay, if that’s what you think let’s bring him back next week, and let’s do a memory test and let’s take it from there”* (GPN 13*).*

#### Sub-theme Funding better dementia care

Australian general practice funding is paid on a fee-for-service basis, with GPs paid for each service they provide. Study participants stated the primary care funding model does not allow for the time required to provide care for PLWD and carer(s) “*because you’re dealing in general practice with an adult in a time slot that is [not]altered for people with dementia or Alzheimer's. We're just expected to care for them in the same timeframe with the same amount of funding as what we get for everybody else. It doesn't work”* (GPN 4).

The GPNs all agreed that the primary care funding model also restricted their ability to involve the carer.* “there should be a Medicare rebate allowing the family member or the caregiver to come for an appointment and sit down, speak to us without having to bring the actual person who the appointment's about with them because it's ridiculous that they have to have that family member in the building for us to see them. We'll quite often have our people cancel appointments because it's not a good day to bring them out. Then what that does to their caregiver mentally, they're needing to come for support and someone to talk to about what's happened, and they've now had to cancel the appointment because they don't want to take their loved one out that particular day. It's just not very supportive”* (GPN 4).

All GPNs believed that funding nurse-led dementia care would facilitate the delivery of best-practice dementia care and that the PLWD and or their carer(s) should be able to “*make an appointment with the nurse. You don’t need to have the doctor involved. The nurse can actually just say these are the steps that we would need to do, where do you think your parent is at, what sort of support work would you like. That’s all pretty easy to do. Then you bring the doctor in as needed rather than the other way around. You can say all right, well, it sounds like we need to make an appointment with mum, I’ll do a 75-year-old assessment, then they’ll see the doctor. I’ll make sure that the doctor’s on board with what you’d like to happen and it might take three or four visits to get this all happening”* (GPN 9).

#### Sub-theme Education, networking and resources

A lack of organisational investment in primary care nurse formal dementia education was perceived as a significant barrier to increasing knowledge and skills. *“I know in the hospitals, often they get paid education days—tell me the last nurse who got paid a paid education day. Why does the practice not just go, okay, our needs are dementia, okay, we're going to send you guys to these courses on these days. But it's because practices, usually they're privately run, they don't get funding from the government to send nurses to those kinds of things so they don't. We don't have time to take time off work to go and seek out education”* (GPN 6). They expressed frustration that there would not be an expectation for other health professionals to work in areas they were not trained for. *“during the appointments you just feel like just out of your depth really, you've got an adult coming in for an issue that you see all the time, but they don't act the same as your adult population. So, I feel like it should almost be categorised like the way that they provide us with specialist training. If you're going into paediatrics, you know that you're going into an environment where things are very different, and you're given that information and that help and support on how to manage. But you're not really given that information, help or support on how to change your behaviour to get what you need and to give what you need to people with dementia”* (GPN 4).

All the GPNs in the study discussed the value of mentoring and networking as effective ways of learning new knowledge and skills and receiving support if they were unsure of what to do. *“I really like the idea of networking and getting people from other practices to share how they change their practice or how they can manage, having support that way. Maybe creating a community of care amongst nurses designed around people caring for dementia. I'm part of a wound care group. So, we meet once a month at a place and discuss different issues in wound care. Having something like that for dementia where you meet up with the other nurses and just have those conversations about what they've struggled within their practice, how they've overcome and that support, I think that would be amazing”* (GPN 3).

All the GPNs wanted easily accessible resources that provided direction on what to do “*it needs to be a bit of a one-stop shop. So, if they do this, if their memory’s 19, okay, this is what you might consider next; what’s the next step? Have you considered their driver’s license? Have you considered support in the home? Even before that, it’s like ticking off advanced care plans, guardianship, power of attorney…obviously it’s going to be like a basic guideline, you’re not going to get everything into it. But just to give you a bit of an idea. Okay, does the GP know? Have you done an MMSE? Then work out a referral pathway from there”* (GPN 11).

### Theme Working collaboratively

#### Sub-theme Different roles, one team

Collaboration both within the general practice clinic and with external agencies was perceived by all GPNs as a facilitator for dementia care. A hierarchical structure with a separation of the GP, GPN and receptionist role, and a perceived lack of trust that the GP will inetegrate the nurse’s findings into their management, compromised the ability of the nurse to provide dementia care. *“we need to be able to work as a team more efficiently … there's the doctor and then there's the nurse and then there's a receptionist after that—and they actually do play a big part in a lot of ways as well. The nurses and doctors [need to] see that we're actually on the same team doing two different parts, and it's not as much of a hierarchy as it is a team. Like, we make our recommendations to the doctor and then we have to trust that the doctor will pass them on and implement them. But often that doesn't happen… if you look at a hospital, if they have a patient who has a difficult, complex case in a hospital, they call a multidisciplinary team together with the family and they sit down and they create a care plan for them. Yes, it is usually a short-term plan to achieve a common goal, but that's what they do. But I guess it would be cool to see us in general practice doing that with the doctor. We might not be able to get the other disciplines in clinic and that's fair, but between the nurses and the GPs to go clinically, GPs, you can order this range of tests and this is necessary and, nurses, we can do this, this and this. That would be cool to see and we would be able to create a more individualised plan for the patients then”* (GPN 6).

#### Subtheme Interagency communication

The lack of shared information between health and community service agencies was seen as a significant barrier to the provision of care to PLWD and their carer(s). “*there needs to be increased interagency communication. The left hand needs to know what the right hand is doing. A little less bureaucracy and a bit more sharing of information, if* it’s *health professional sharing information, it should be allowable. You’re not gossiping"* (GPN 1).

The GPNs reported that the lack of communication with other agencies involved in a patient’s care led to poor care continuity and difficulty in ensuring the PLWD and carer(s’) needs were being met. *“if I can [find] out [from the community care provider] what’s happening. Then if there [are] holes and cracks, when I see that patient next time, I can go, oh okay. I see that this, this and this has been done. Okay, what’s happening now? Something else has happened. Okay, we need to sort that and you might need this. Then get them back in and add another referral in. Whereas now they’re coming back and you don’t know what’s happened or what has been done”* (GPN 11). One GPN reported how good collaborative relationships with other care providers facilitated provision of care *“There’s two homecare providers in town that I’ve actually got very good rapport with. They let me know if there’s issues. If there’s issues, something like, you know, they’re not taking [their medications] the message gets back to the homecare support supervisor who gets in touch with me and we work out what happens next”* (GPN 12).

## Discussion

The emphasis on care for PLWD and their carer(s) is increasingly focussed on supporting people and families to live as well as possible with dementia across the disease trajectory [32]. Optimising the role of GPNs has the potential to deliver best-practice dementia care in the primary care setting where dementia is reluctantly disclosed, poorly recognised, under diagnosed, and less than optimally managed [1]. In this study, the GPNs thought that the five published best-practice dementia care recommendations contained in the Australian Clinical Practice Guidelines and Principles of Care for People with Dementia [13] as best-practice dementia care and highly relevant to the role of the GPN [14] were too vague to easily put into practice. The study participants reported that they considered all five of the recommendations were important in delivering care to people living with dementia. The study participants reported multiple barriers and facilitators to their implementation.

As reported in previous studies, the GPNs in this study strongly believed that they were better suited than the general practitioner to provide care for PLWD and their carer(s) and should be included in a team-based approach to care [11]. All study participants perceived their approach to care as holistic [11, 33] based on building therapeutic relationships, which is well established as essential to the delivery of person-centred dementia care [34, 35]. This approach to care underpins all five best-practice recommendations under investigation in this study [14]. The strong belief they could support PLWD and their carer(s) was perceived as a significant facilitator to the implementation of best practice dementia care recommenndations into daily practice by the GPN.

Further facilitators to the implementation of the best-practice dementia care recommendations identified by the GPNs in this study included being proactive in having a conversation about dementia, integrating dementia into other chronic disease management care delivery, and taking a dyadic approach to care. These elements of care are supported in the literature as essential in meeting the health and social care needs of both the PLWD and the carer(s). Stigma, fear and denial are significant barriers to people concerned about their cognition and or families seeking out information and support [36]. The use of effective and thoughtful communication by GPNs can potentially mitigate stigmatizing beliefs and stereotypes associated with dementia and encourage open conversations [37]. Findings of studies in primary care suggest that supporting the PLWD without due consideration of the carer(s) can result in poorer health outcomes for both [3, 38]. GPNs already have a well-established and accepted role in chronic disease management [39]. Given that it is common for PLWD to have at least one other long-term health condition [32, 40] and higher rates of hospitalisation compared with people living without dementia [41], high-quality care needs to address the impact of dementia in all chronic disease management interventions.

All GPNs in this study described a lack of knowledge and skills as a barrier to provision of best-practice dementia care, particularly in recognising potential dementia, initiating a conversation about cognition and knowing what services were available to support PLWD and their carer(s). Poor knowledge and skills is well recognised as a barrier to dementia care provision; however, few formal education programs target GPNs [42] and it is known that translation of education, particularly as a single activity, to clinical practice is poor [3, 41, 43]. In this study GPNs highly valued access to peer support through informal networking, mentorship, and formal and informal access to specialist support as opportunities to improve knowledge, skills and confidence. This was, in part, due to the reported barriers to accessing formal education, including lack of funding, time and organisational support. It is known that networking and mentoring can facilitate communication and knowledge sharing between nurses and offer opportunities for peer support [41] and, with the increasing acceptability and use of technology-based meetings, may be a more flexible and accessible alternative to formal training programs, particularly for sole practitioners and those working in rural and remote healthcare settings [44]. However, the design and implementation of networking strategies, such as communities of practice, that effectively support information sharing and knowledge transfer is not well understood [44].

All the GPNs in this study reported a need for easily accessible resources, including guidelines, checklists and care pathways, to inform best-practice dementia care. To our knowledge there are limited resources available targeting GPNs. It is also well-established that guidelines do not always impact clinical practice change [16]. Care pathways have been described in the literature as a process to develop and implement well-organised care and improve quality and efficiency [45]. Effective care pathways depend on enhanced team work [45] and a lack of team work was identified by all GPNs as a significant barrier to best practice dementia care. This suggests that general practice team culture change would be needed as part of the developmemt and implementation of dementia care pathways.

Environmental contexts, including primary care funding models, the internal general practice team culture, limited open communication with external agencies and a largely part-time nurse workforce presented significant barriers to GPNs providing best-practice dementia care. Nurses employed in general practice in the main work for either small businesses or corporate chains and do not generate income unless working under the direction of the general practitioner. Research has shown that GPNs do not routinely collaborate in patient care and decision-making with the dotor but are delegated tasks that generate income for the practice [46]. The GPN usually has to rely on the doctor to follow through on the GPN’s recommendations for care which, as reported in this study, often does not happen. This lack of professional autonomy and status and a medical practitioner activity-based funding model further entrench the hierarchy, and as described by nurses in this study, result in the nurses feeling demoralised and disincentivised, presenting a significant barrier to provision of best-practice dementia care. GPNs described the development of trusting practitioner-patient relationships and continuity of care as important in the delivery of person-centred dementia care. However the study participants described themselves as a largely part-time workforce which impeded contiunty of care. Authors of the Australian Primary Care Nurse workforce survey (2019) [47] reported that 49.6% of survey respondents worked part-time.

### Strengths and limitations

A strength of this study was the use of semi-structured interviews to facilitate primary care nurses to contribute, explore and clarify their views on a research topic that little is known. Another strength was the structured analysis process. The high level of agreement between the themes generated independently by the authors increase our confidence in the results.

The use of a convenience sample can decrease trustworthiness. However, including an experienced group of primary care nurses, the majority of which had some degree of dementia training, meant they could actively reflect on and provide insightful responses to the research questions, enriching the findings about their role in the care of people living with dementia.

A limitation was the small sample size as it is likely not representative of all GPNs, however there was considerable consistency in responses. Not all states and territories of Australia were represented in the study population, with no GPNs from the Australian Capital Territory and Northern Territory and Tasmania included. However, as shown in the Australian Primary Care Nurse workforce survey (2019) [47] these three regions of Australia comprise only 9.4% of the primary location of place of work for Australian GPNs. Similarly, no males participated in the study. In the Australian Primary Care Nurse workforce survey (2019) data [47] 96.1% of the workforce was female and only 3.8% were male.

### Implications of study findings

As the number of PLWD and their carer(s) increases, the GPN role in dementia care provision within the primary care team needs to be optimised. Theoretical frameworks for the development and operationalisation of complex interventions describe. understanding barriers and enablers as an essential initial step. The knowledge gained in this study can potentially inform the development of multi-faceted interventions to support the GPN provision of best practice dementia care.

## Conclusion

This study identifies a number of practitioner and organisational level factors that impact on GPNs ability to integrate best-practice dementia care recommendations into daily clinical practice. Strategies to mitigate these barriers and enhance the facilitators that GPNs perceive prevent the delivery of best-practice care for PLWD and their carer(s) need to be implemented.

## Data Availability

The transcripts analysed in this study are available from the corresponding author on reasonable request.

## Abbreviations

GP: General Practitioner
GPN: General Practice Nurse
PLWD: Person/ People living with Dementia
